# Unravelling Vanillin Biosynthesis: Integrative Transcriptomic and Metabolomic Insights into Pod Development

**DOI:** 10.1021/acs.jafc.5c05293

**Published:** 2025-07-17

**Authors:** Rebeca Hernández-Peña, José Luis Lorenzo-Manzanarez, Luis Alfredo Cruz-Ramírez, Delfino Reyes-López, Carmela Hernández-Domínguez, Fermín Pascual-Ramírez, José J. Ordaz-Ortiz

**Affiliations:** † Metabolomics and Mass Spectrometry Group, Unidad de Genómica Avanzada, Centro de Investigación y de Estudios Avanzados del Instituto Politécnico Nacional, Carretera Irapuato-Léon, Km. 9.6, Libramiento Norte, C.P. 36824 Irapuato, Guanajuato, Mexico; ‡ Molecular and Developmental Complexity Group, Unidad de Genómica Avanzada, Centro de Investigación y de Estudios Avanzados del Instituto Politécnico Nacional, Carretera Irapuato-Léon, Km. 9.6, Libramiento Norte, C.P. 36824 Irapuato, Guanajuato, Mexico; § Facultad en Ciencias Agrícolas y Pecuarias, 3972 Benemérita Universidad Autónoma de Puebla, San Juan Acateno, Teziutlán, C.P. 73965 Puebla, Mexico; ∥ Instituto de Investigaciones en Ecosistemas y Sustentabilidad, UNAM, Campus Morelia, C.P. 58190 Morelia, Michoacán, Mexico

**Keywords:** benzoate, ferulate, LC–MS, nontargeted metabolomics, phenylpropanoids, RNA-seq, targeted metabolomics

## Abstract

Vanilla is the most popular flavor in the world and the second most valuable spice after saffron. Vanillin (4-hydroxy-3-methoxybenzaldehyde) is the predominant compound in natural vanilla flavor, a complex mixture comprising over 200 compounds. However, its biosynthetic pathway remains unclear. This study presents a comprehensive transcriptomic and metabolomic analysis of leaves and pods throughout development, providing insights into vanillin biosynthesis. Differential gene expression analysis identified key genes involved in the ferulate and benzoate pathways, while metabolomic profiling revealed stage-specific accumulation patterns of pathway intermediates. Vanillin levels peaked 6 months after pollination, followed by a steady increase in the glucoside form. This integrative omics approach suggests a coordinated regulation of both pathways during fruit development, offering valuable insights into the metabolic dynamics of vanillin production and establishing a foundation for future research on genetic and metabolic engineering strategies in .

## Graphical abstract

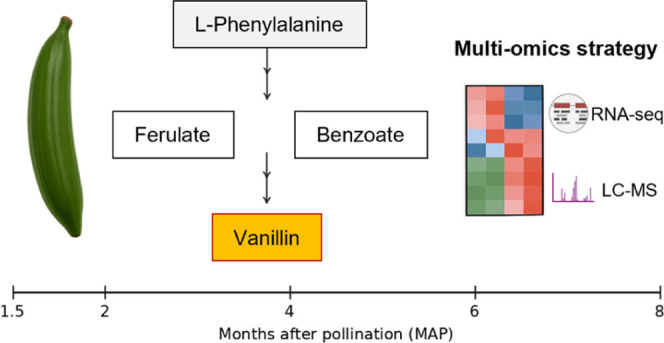

## Introduction

The monophyletic genus belongs to the Orchidaceae family and comprises approximately 121 pantropical species.^1^ Although some of these species, mainly of American origin, possess aromatic qualities, only three have significant economic value globally and for local small-scale production: , and . The first is the most cultivated for commercial production due to its superior aroma and flavor quality.^2^

 is native to the tropical forests of Mesoamerica and has been an integral part of traditional Mexican culture since pre-Hispanic times.^3^ This perennial hemiepiphytic orchid features adventitious aerial roots that provide lateral support. The flowers are diurnal and bisexual but cannot self-pollinate due to the *rostellum*, a membrane that physically separates the reproductive structures.^4^ Pollination is conducted manually because of the lack of natural pollinators. Moreover, the stem-cutting propagation method has led to a loss of genetic diversity.^5^ Vanilla fruits, harvested 8–9 months after pollination (MAP), are seed capsules commonly called pods or beans once cured. They lack aroma because the primary aromatic constituents are stored as glucosides, making the curing process of green fruits essential.^6^

Vanilla is the most popular flavor in the world and the second most valuable spice, following saffron. The natural extract from is considered superior and more desirable than synthetic alternatives, making up approximately 1% of the total vanilla market. The challenges in production have created a high demand for this premium product, resulting in several studies on , the primary source of this valuable and increasingly sought-after spice.^2^

Vanillin (4-hydroxy-3-methoxybenzaldehyde) is the predominant compound found in natural vanilla flavor, a complex mixture comprising over 200 compounds;^7,8^ it makes up as much as 2.5% of the dry weight (DW) of cured pods.^9^ Its aldehyde group is highly reactive and can be toxic to cells at high concentrations. For this reason, it is stored in its conjugate glucoside form within specialized cellular compartments referred to as phenyloplasts.^10^ Like lignin and benzoic acids, vanillin is a product of phenylpropanoid metabolism, while phenylalanine is widely recognized as the primary precursor; the involvement of tyrosine in the conversion of proposed intermediates has also been documented.^11^ According to various proposed biosynthetic pathways, vanillin is derived from *trans*-cinnamic acid and is then converted to coumaric acid by the action of cinnamate 4-hydroxylase (C4H) and cytochrome P450 reductase (POR).^9,12^ The conversion of coumaric acid to vanillin involves the following main steps: side chain shortening, introduction of a side chain aldehyde function, addition of a 3-hydroxyl group, and 3-*O*-methylation. Determining the precise sequence of these reactions has proven challenging, and significant uncertainties still exist during the chain-shortening step.^9,13^

Several vanillin biosynthetic pathways have been proposed, but two main hypotheses exist: the first suggests that vanillin is formed from monomeric lignin precursors. This pathway, referred to as “Ferulate”, involves the hydroxylation and methylation of 4-coumaric acid, followed by side-chain reduction of ferulic acid.^14,15^ The second hypothesis, known as “Benzoate”, begins with the chain shortening of 4-coumaric acid to 4-hydroxybenzaldehyde, and continues to the hydroxylation and methylation of the aromatic ring.^16–18^

Numerous mechanisms for chain shortening in plants have been described, including two cytosolic nonoxidative pathways (CoA-dependent and CoA-independent) and a β-oxidative pathway in peroxisomes.^19^ While the cytosolic pathways are not well understood, the β-oxidative pathway has been studied in flowers.^20^ This pathway resembles fatty acid degradation and shortens the propyl side chain through three enzymatic steps,^21^ with 3-ketoacyl-CoA thiolase catalyzing the formation of benzoyl-CoA (BA-CoA), the first C6–C1 intermediate. Using petunia flowers, which produce high levels of benzenoid/phenylpropanoid compounds, as a model system, Adebesin et al.^22^ showed that downregulation of a thioesterase (*PhTE1*) results in accumulation of BA-CoA and a reduction in the size of the BA pool. Along with the increased formation of benzylbenzoate and phenylethyl benzoate, these data indicate that the product of the core BA β-oxidative pathway, BA-CoA, can be exported from the peroxisomes. Moreover, that thioesterase downregulation leads to increased production of volatile phenylpropenes (eugenol, isoeugenol, and vanillin were increased by up to 360%, 220%, and 280%, respectively), lignin, and anthocyanins, suggesting that the ratios of peroxisomal acyl-CoAs to free CoA influence the distribution of carbon flux between the BA β-oxidative and phenylpropanoid pathways.

The resolution of the actual pathway study of this mechanism has been hindered by the general promiscuity of the enzymes involved in the metabolism of phenolic compounds, the challenges in interpreting labeling experiments,^13^ and, although a method for transformation and gene editing has been reported,^23^ no functional validation related to vanillin biosynthesis has been conducted to date.

The applications of omics technologies in spice crops are extensive. By measuring various biological components, such as gene expression, protein levels, and metabolites, researchers have gained insights into plant regulation mechanisms and responses, as well as their spatial and temporal dynamics, particularly in medicinal plants.^24^ However, in , the lack of robust genetic systems has impeded progress in understanding vanillin biosynthesis. Recent studies have published genomics-based diversity analyses of the genus.^25–27^ These analyses were followed by the release of a chromosome-scale genome for , which revealed gene variants influencing fruit quality and productivity.^28^ Additionally, partial endoreplication was detected through a precise haplotype-phased genome.^29^ Furthermore, transcriptome sequencing has enabled the structural and functional exploration of various biological processes, including gene expression profiles, biomarker identification, and gene discovery and isolation. Three RNA-seq data sets for have been published. One data set focuses on a tissue-specific transcriptomic analysis of fruit development, identifying candidate genes involved in the monolignol pathway and their temporal expression patterns.^30^ The second data set examines transcriptional changes during the early stages of infection.^31^ Lastly, the most recent transcriptome provides a comparative approach between two accessions with differential vanillin content, enabling the identification of candidate genes alongside key positive and negative regulators that influence vanillin accumulation.^32^ However, due to the biological complexity of , generating a new transcriptome is essential to comprehensively capture gene expression patterns and regulatory networks that were not addressed in previous studies, thus facilitating further functional and comparative analyses.

A plant phenotype can also be characterized by its metabolites. Previous research on has compared the metabolomic variations in green^33–35^ and cured pods^7,33,36^ using various techniques. Over time, the diverse approaches proposed for vanillin biosynthesis raise the possibility that specific systems, environments, or conditions may require different mechanisms. Nonetheless, examining which pathway is primarily followed and which holds potential benefits in metabolic engineering is imperative.^37^ Very little research has explored the potential for elucidating the biosynthesis of the vanillin pathway by combining different omics approaches. Herein, we report a comprehensive analysis of the transcriptome and metabolome of leaves and pods during development, providing evidence that enhances our understanding of vanillin biosynthesis.

## Materials and Methods

### Chemicals and Reagents

Unless otherwise stated, all chemicals and reagents were sourced from AccesoLab (AccesoLab S.A. de C.V., Mexico City, Mexico). Ultrapure grade water was acquired from a Milli-Q Gradient A10 system (Merck-Millipore). Methanol, acetonitrile, and formic acid were MS-grade from Sigma-Aldrich (Merck S.A. de. C.V., Mexico City, Mexico), along with analytical standards with a purity of ≥99% as indicated by the supplier.

### Sample Collection

 flowers were hand-pollinated and labeled at the Benemérita Universidad Autónoma de Puebla’s vanilla germplasm bank in April 2021. Three pods and four leaves were collected from five Mexican accessions and immediately frozen in liquid nitrogen. The collected tissues were divided in half for transcriptomics and the other half for metabolomics analysis. They were then stored at −80 °C until further use. Sampling was conducted five times after pollination (1.5, 2, 4, 6, and 8 months, MAP) (Figure 1). Materials for each accession were collected at various stages by using the same vanilla plant. A total of 50 samples were obtained (5 accessions × 5 time points × 2 tissues). Each vanilla plant represents a biological replicate.

**Figure 1:**
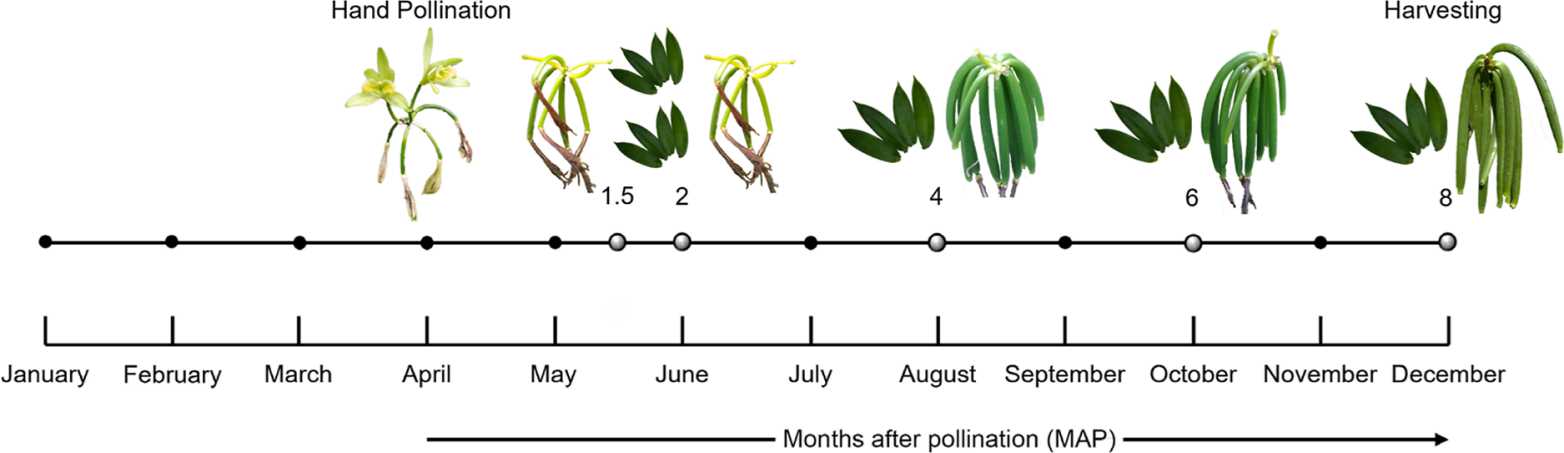
Sampling timeline for five biological replicates of pods and leaves at the Mexican germplasm bank. Gray circles indicate the sample collection dates at 1.5, 2, 4, 6, and 8 months after pollination (MAP).

### RNA Extraction, Library Construction, and RNA Sequencing

Leaves and pods were ground into a fine powder with liquid nitrogen. Total RNA was extracted from 250 mg of frozen tissue using an Invitrogen TRIzol Plus RNA Purification Kit, with slight modifications to the provider’s protocol. RNA concentration and quality were measured by a Nanodrop ND-1000 spectrophotometer (Thermo Fisher Scientific, Wilmington, DE, USA) and a Bioanalyzer 2100 (Agilent Technologies, Santa Clara, CA, USA), respectively. RNA integrity number values of all samples were above eight. Fifty libraries were constructed for 150 pb paired-end sequencing on the MGISEQ-2000 platform using DNBSEQ technology.

### Transcriptome Data Analysis

The quality of RNA-seq data was assessed using FastQC v0.11.7.^38^ The trimming process was carried out with Trimmomatic v0.38,^39^ and the clean reads were mapped to a reference genome (unpublished data), which was generated from a clone of the same germplasm bank collection, utilizing HISAT2 v2.1.0.^40^ Aligned read counts were computed using HTSeq v0.11.2.^41^ Differentially expressed genes (DEGs) were evaluated by contrasting neighboring times of the same tissue and the same time in different tissues, employing the Bioconductor package edgeR,^42^ based on a false discovery rate (FDR) < 0.05 and absolute log_2_ (fold change) ≥ 2.

The proteins predicted from the unigenes were compared with a reference database using the BLASTp algorithm (with an *e*-value cutoff of 10^–5^).^43^ For Gene Ontology (GO) enrichment analysis, the unigenes were mapped using the ShinyGo program v0.81 with -like reference species. A 0.05 statistical significance threshold FDR was applied.^44^ The top ten unigenes for each of the GO terms were listed. The unigenes were searched for enriched signal transduction pathways in the Kyoto Encyclopedia of Genes and Genomes (KEGG) database. For the GO terms and KEGG pathway analysis, we used a *q*-value < 0.05.^45^

### Metabolite Extraction

Frozen leaves and pods were initially freeze-dried and then ground into a fine powder. Two hundred milligrams of powder were extracted with 1 mL of hexane. Each sample was agitated in a vortex and then centrifuged at 1000*g* for 20 min. The supernatant was discarded. Subsequently, 1 mL of 80% aqueous methanol acidified with HCl was added (pH 1.5). Samples were agitated for 5 h at room temperature and centrifuged at 1000*g*. The supernatant was recovered after 20 min, and this procedure was repeated twice. The extract was neutralized to pH 7 with NH_4_OH and dried under vacuum in a miVac Duo Concentrator (Genevac).

Before LC–MS analysis, the samples were resuspended in 50% aqueous methanol, and 1.5 mL of the extract was passed through a 0.2 μm PTFE filter (Agilent Technologies, USA). An aliquot of the same volume for all extracts was used to prepare five quality control (QC) samples. Extracts obtained from each sample were used for nontargeted and targeted metabolomic analyses.

### Nontargeted Metabolomic Analysis

Samples were analyzed using a UPLC-ESI-Q-TOF-MS system (Acquity I-class, SYNAPT G1 HDMS, Waters Corporation). Chromatographic separation was performed on a reversed-phase CSH C18 column (2.1 × 150 mm; 1.7 μm, Waters Corporation) at 35 °C. Mobile phase A consisted of ultrapure water containing 0.1% formic acid (v/v), while mobile phase B comprised acetonitrile with 0.1% formic acid (v/v) at a flow rate of 0.3 mL/min. The gradient elution conditions were as follows: isocratic 1% B for 1 min; B increased linearly to 47% from 1 to 16 min; then B increased linearly to 99% from 16 to 18 min and was maintained for 3 min to wash the column; finally, isocratic 1% B was used for 3 min to re-equilibrate the column. The mass spectrometer range was set from 50 to 1200 Da. In positive electrospray ionization (ESI) mode, the conditions were as follows: capillary voltage 1.8 kV, cone voltage 40 V, collision energy 10 eV (function 1, Low energy) and ranging from 20 to 50 eV (function 2, high energy), source temperature 110 °C, desolvation temperature 250 °C, and desolvation gas flow 500 L/h. In negative ESI mode, the conditions were similar except for the desolvation temperature, which was 200 °C.

The MS system was calibrated with sodium formate. Leucine enkephalin (2 ng/mL) was the lock mass compound infused at a flow rate of 6 μL/min flow rate. Data acquisition was performed in continuum mode with a scan time of 1.5 s. QC samples were analyzed every 10 injections to evaluate the stability of the LC–MS system.

### Nontargeted Metabolomic Data Analysis

Data alignment, normalization, peak picking, and deconvolution were conducted using Progenesis-QI for metabolomics software (v2.4, Nonlinear Dynamics, Waters Corporation, Milford, MA, USA). Compound preidentification was accomplished using PlantCyc, HMDB, ChEBI, and an in-house built database.^46–48^ Preannotated compounds obtained in both ESI modes were compiled into a single matrix of relative intensities. The normalized data were Pareto scaled and utilized for principal component analysis (PCA), clustering heatmap visualization, and pathway analysis using the database of KEGG in the MetaboAnalyst 6.0 web-based platform.^49^

### Targeted Metabolomic Analysis of Vanillin Biosynthesis Pathway-Related Compounds

Eight compounds associated with the vanillin biosynthesis pathway were selected and quantitated using the external standard method across all samples. *Trans*-cinnamic acid, 4-coumaric acid (*p*-coumaric acid), caffeic acid, ferulic acid, 4-hydroxybenzaldehyde, and 3,4-dihydroxybenzaldehyde were analyzed by UPLC-ESI-Q-TOF-MRM (Acquity I-class, SYNAPT G1 HDMS, Waters Corporation) in negative ionization mode. A BEH C18 column (2.1 × 50 mm; 1.7 μm, Waters Corporation) was maintained at 35 °C. The same mobile phases used in the nontargeted analysis were employed, with a flow rate of 0.2 mL/min. A 5 μL sample injection was performed, and the following gradient program was implemented: a 10% B isocratic step for 7 min; then, from 7 to 12 min B was increased linearly to 40%; a washing isocratic step was established from 12.1 to 13 min at 99% B; and finally 10% B was applied for 2 min for column re-equilibration. Source conditions included a capillary voltage of 1.8 kV, a cone voltage of 40 V, a source temperature of 100 °C, a desolvation temperature of 150 °C, a cone gas flow of 20 L/h, and a desolvation gas flow of 500 L/h. The instrument was operated in enhanced duty cycle (EDC) mode. A specific ion for each analyte was filtered in the quadrupole, fragmented in the collision cell, and orthogonally accelerated in the pusher. The [M – H]^−^ ions of analytes were selected as precursors and fragmented with optimized collision energy. The product ions were utilized for EDC, and their respective signals were employed to determine the analytes (Table S1). Mobile phase A was used to prepare five calibration solutions that spanned a specific concentration range for each analytical standard (Table S1). MassLynx (v4.1, Waters Corporation) was used for peak integration and quantitation based on calibration curves against the corresponding standard. The limits of detection (LOD) and limits of quantitation (LOQ) were calculated as LOD = 3.3 s/m and LOQ = 3 LOD, where *s* is the standard deviation of the blank (with five runs) and *m* is the slope of the related analytical standard curve (Table S1).

Vanillin and vanillin glucoside (Vanillin 4-*O*-β-d-glucoside) (ChemCruz; Santa Cruz Biotechnology) were quantitated using HPLC-DAD (Agilent 1200 series). A ZORBAX Eclipse Plus phenyl–hexyl column (4.6 × 150 mm; 5 μm, Agilent Technologies) was employed and maintained at a temperature of 40 °C. Solvent A consisted of ultrapure water containing 0.025% trifluoroacetic acid (v/v), while solvent B was 100% acetonitrile. The flow rate was set at 1 mL/min, and a sample volume of 5 μL was injected. The gradient program was isocratic at 20% B for 1 min, then increased linearly to 35% from 1 to 7.8 min; an isocratic 90% B washing step was implemented from 8 to 10.5 min; finally, isocratic 20% B was employed for column re-equilibration over 2.5 min before the next injection. Eight calibration solutions were prepared in mobile phase A, spanning a concentration range from 1 to 75 μg mL^–1^. Spectrophotometric detection was conducted at 280 nm for vanillin and its glucoside; 600 nm was used as the reference wavelength to enhance the chromatographic baseline. Each sample was analyzed in five independent replicates. ChemStation software (Agilent Technologies) was utilized for peak integration and quantitation. Moreover, the LOD and LOQ were calculated as previously described (Table S1).

## Results

### Global Transcriptome Data between Tissues and Developmental Stages

To understand the gene expression profile of during development, 50 cDNA libraries were paired-end sequenced, corresponding to five biological replicates from five time points and two different tissues. On average, the size of the libraries varied between 20.1 million reads per sample in leaves and 21.9 million in pods. After filtering and trimming, the percentage of preserved reads ranged from 92.7% in leaves to 95.1% in pods. The clean reads were mapped to an in-house genome assembly (unpublished data; Table S2). Gene abundances were estimated from the mapped reads and used in subsequent differential expression analysis. Additionally, to obtain a representative transcript for each gene, regardless of splicing variants, the most extended transcript was designated as the unigene for each gene.

### Identification of DEGs and Their Functional Enrichment Analyses

To describe the functional enrichment in pod tissues, we analyzed the up-regulated and down-regulated genes by comparing pairs of consecutive developmental stages (Figure 2a). Subsequently, GO enrichment (Figure 2b and Table S3) and KEGG pathway analyses (Figure 2c) were performed for the up-regulated DEGs.

**Figure 2:**
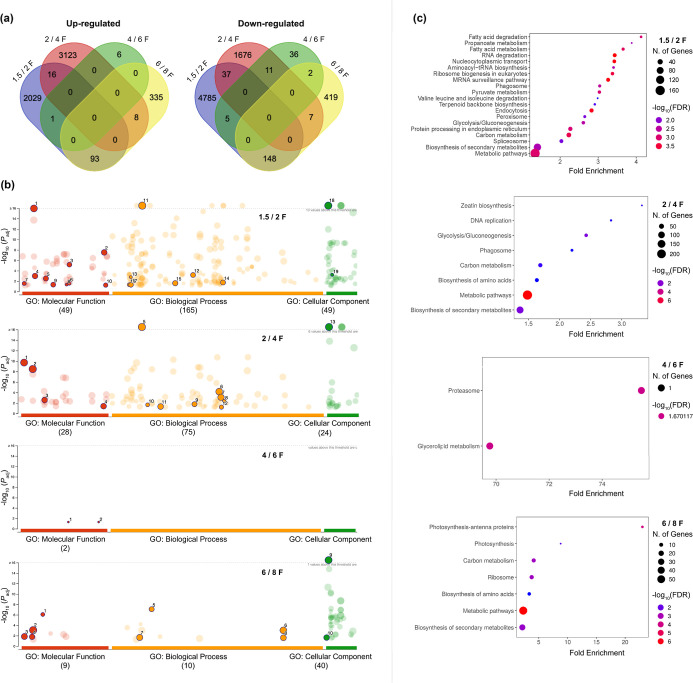
Transcriptomic profiling of pod development. (a) Venn diagram illustrating the overlap of DEGs during fruit development, including up-regulated and down-regulated genes across 1.5/2F, 2/4F, 4/6F, and 6/8F MAP stages. The numbers and circles depict the unique or overlapping DEGs that share regulatory patterns across various stages. (b) GO enrichment analysis of DEGs based on transcriptomic data from pods at 1.5/2F, 2/4F, 4/6F, and 6/8F MAP. The size of the circles indicates the number of genes overrepresented in specific categories of molecular function (MF), BP, and cellular component (CC) in relation to GO. The numbers correspond to the IDs of the biological functions, which are detailed in Table S3. The Benjamini–Hochberg method was employed to adjust the *P* value (*P*_adj_) for multiple testing. (c) KEGG pathway analysis of enrichment for DEGs up-regulated in pods at 1.5/2F, 2/4F, 4/6F, and 6/8F MAP. Fold enrichment indicates the percentage of genes associated with a specific pathway. The FDR illustrates the likelihood that the enrichment is due to chance.

In pods, 12,737 DEGs were identified, with 5611 transcripts up-regulated and 7126 down-regulated. In the 1.5/2F MAP comparison, 2029 transcripts were up-regulated (Figure 2a). The most enriched categories for MF included “protein binding” (1), “ATP-dependent activity” (2), and “translation regulator activity” (3). For the BP, the predominant categories were “cellular process” (11), “ribosome biogenesis” (12), and “vacuolar protein processing” (13). In contrast, the cellular component (CC) consisted of “intracellular anatomical structure” (18) and “vacuolar proton-transporting V-type ATPase” (19) (Figure 2b). KEGG pathway analyses indicated that “metabolic pathways”, “biosynthesis of secondary metabolites”, and “spliceosome” were the most relevant (Figure 2c). For the 2/4F MAP comparison, 1676 up-regulated transcripts were detected (Figure 2a). The most enriched MF categories were “catalytic activity” (1), “binding” (2), “hydrolase activity” (3), and “ATP-dependent activity” (4). In BP, “cellular process” (5), “response to stimulus” (6), and “localization” (7) were predominant, while for CC, “intracellular anatomical structure” (13) was the most enriched (Figure 2b). KEGG analysis identified “biosynthesis of secondary metabolites”, “protein processing in endoplasmic reticulum”, and “endocytosis” as key pathways (Figure 2c). In contrast, the 4/6F MAP comparison revealed only six up-regulated transcripts (Figure 2a), with MF enrichment limited to “galactolipid galactosyltransferase activity” (1) and “beta, beta digalactosyldiacylglycerol” (2) (Figure 2b). KEGG pathway analyses identified “biosynthesis of secondary metabolites”, “metabolic pathways”, and “biosynthesis of amino acids” as the most relevant pathways (Figure 2c). Finally, for the 6/8F MAP comparison, 335 up-regulated transcripts were found (Figure 2a). The most enriched MF terms included “chlorophyll binding” (1), “binding” (2), and “mRNA binding” (3). The most relevant BP categories were “photosynthesis” (5), “organonitrogen compound metabolic process” (6), and “response to abiotic stimulus” (7). For CC, the key terms were “cytoplasm” (9) and “plant-type vacuole” (10) (Figure 2b). KEGG pathway analysis highlighted “biosynthesis of secondary metabolites”, “metabolic pathways”, and “biosynthesis of amino acids” (Figure 2c).

Similarly, DEGs in leaf tissues were analyzed between pairs of consecutive developmental stages (Figure S1a). GO enrichment analysis (Figure S1b and Table S4) and KEGG pathway analysis (Figure S1c) were subsequently conducted for the up-regulated DEGs.

### Mapping of Transcripts Related to the Biosynthetic Pathway of Vanillin

Considering the genes encoding the enzymes reported in the ferulate and benzoate pathways, we selected 32 and 8 of our transcripts, respectively. The relative abundance of these transcripts was assessed in each pair of consecutive pod development stages. These transcripts were then mapped to the CR0040 chromosomes^29^ (Figure 3).

**Figure 3:**
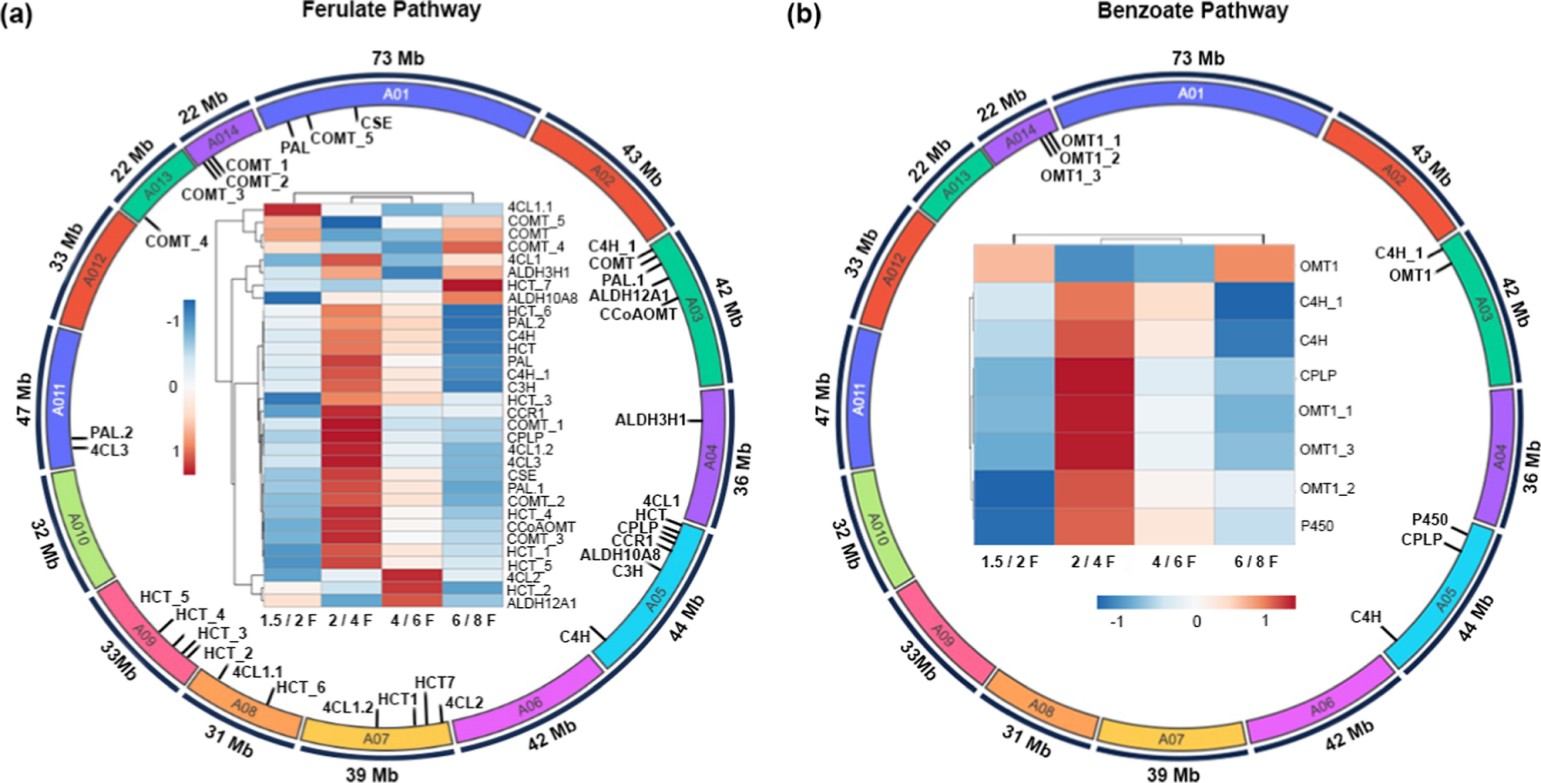
CR0040 chromosome-level mapping of the genes putatively involved in the ferulate and benzoate pathways. The heatmap represents the transcript abundance of genes related to (a) ferulate and (b) benzoate pathways across the analyzed pod developmental stages. The heatmap color gradient represents the relative sequence abundance. Numbers in the color key indicate log_2_ FC. The color in the circle indicates the chromosome number, and the black line shows the localization of the gene coding for the enzymes involved in each pathway. The size of each arc is represented in Mb.

### Metabolite Dynamics at Different Developmental Stages

A total of 50 extracts were analyzed using a nontargeted metabolomic approach. In the positive and negative ionization modes, 627 and 809 features were detected, respectively. Seven hundred seventy-eight of these features were preidentified with a match score of over 80% based on the exact mass of the precursor ions (*m*/*z*), the corresponding fragmentation spectra, and the precursor isotopic pattern. The data were combined into a single matrix (Table S5), which included the PubChem ID, description, ionization mode, and average relative abundance per sample. The most abundant classes in immature pods and leaves were glucosides, lipids, phenylpropanoids, organic acids, and organoheterocyclic compounds. In contrast, the predominant classes in mature pods included phenylpropanoids, lignans and neolignans, benzenoids, alkaloids, and organic oxygen compounds. A 2D PCA with an explained variance of 60.3% based on the first two components (*R* ^2^ 0.950) (Figure 4a), demonstrating a clear separation between immature (1.5 and 2 MAP) and mature pods (4, 6, and 8 MAP). Meanwhile, leaf samples were indistinguishable by the developmental stage. Furthermore, the QCs were positioned in the center of the plot, indicating good reproducibility, as they were arranged together in the unsupervised test. This pattern aligned with the hierarchical clustering analysis presented in Figure 4b. The samples were clustered into two major clades: the right clade comprised pods, highlighting that each developmental stage has characteristic compounds that allow for their distinction; the left clade contained leaves that could not be grouped by developmental stage, suggesting that the metabolites remained unchanged during leaf development. Immature pods and leaves share some compounds associated with primary metabolism. Figure 4c displays the heat map of the abundance-scaled 70 top discriminant preannotated compounds in pod samples grouped by MAP. The dendrogram on the left clustered the features; the upper clade corresponded to compounds found in mature pods, while the lower clade represented those in immature pods.

**Figure 4:**
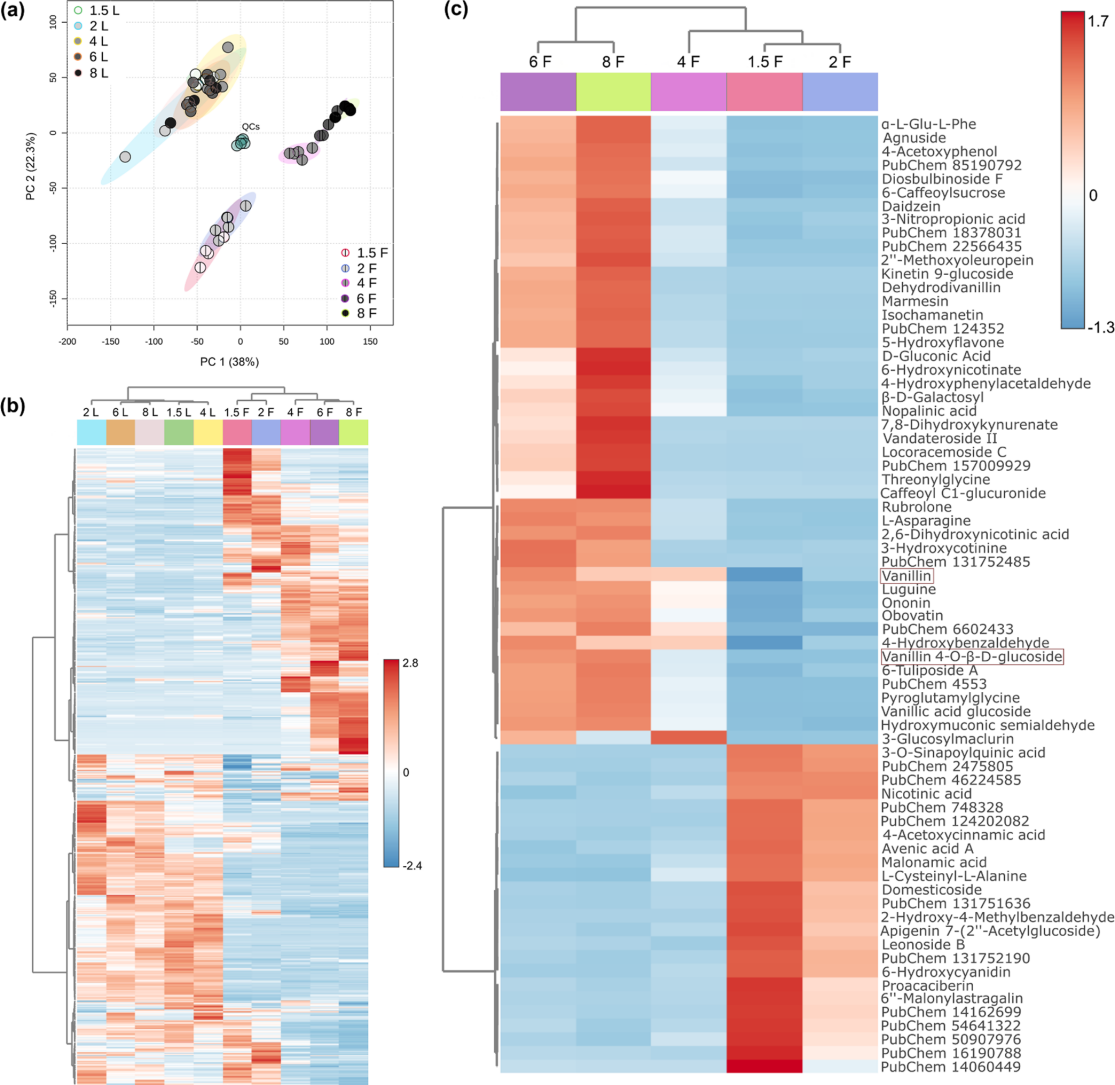
PCA and heat map visualization of the leaves and pods of at various developmental stages, based on five biological replicates. L denotes leaves; F denotes fruits; 1.5, 2, 4, 6, and 8 MAP. (a) PCA includes QCs, *x*-axis principal component 1 (38%), and *y*-axis principal component 2 (22.3%); *R* ^2^ 0.950. Temporal progression is illustrated with a gray to black color scale. (b) Heat map of hierarchical clustering analysis using Euclidean distance and Ward’s linkage method. (c) Heat map of the top 70 preannotated compounds in fruit samples. A description of each ID is provided in Table S5.

Pathway analysis was also conducted using preannotated compound identifications from pod samples. Based on the impact and *p*-value < 0.05, phenylpropanoid biosynthesis, carbon fixation, and the metabolism of compounds such as alanine, aspartate, glutamate, nicotinate, nicotinamide, glycine, serine, threonine, tyrosine, starch, and sucrose were significantly represented (Figure S2).

### Targeted Analysis of Compounds Related to the Vanillin Biosynthetic Pathway

The quantitation of vanillin, vanillin glucoside, and six compounds associated with the biosynthetic pathways for vanillin was conducted in the 50 samples using the same extracts from the nontargeted analysis, with the aid of analytical standards (Table S6). A comparison was made between the oxidative route of ferulate and the nonoxidative benzoate pathway. The precursors shared by the two pathways were *trans*-cinnamic and 4-coumaric acid. Given the commercial availability of analytical standards for the ferulate route, only caffeic and ferulic acids were quantitated, while 4-hydroxybenzaldehyde and 3,4-dihydroxybenzaldehyde were selected for the benzoate hypothesis.

Vanillin was detected only in pod samples from the second month of development, reaching its highest concentration in the sixth month and declining toward the harvest period. In contrast, the accumulation of vanillin glucoside significantly increased during the pod’s ripening, with the highest concentration observed at eight MAP (Figure 5a). *Trans*-cinnamic acid, the first common precursor of both pathways, exhibited a declining pattern, while 4-coumaric acid increased, becoming more abundant at six MAP (Figure 5b,c).

**Figure 5:**
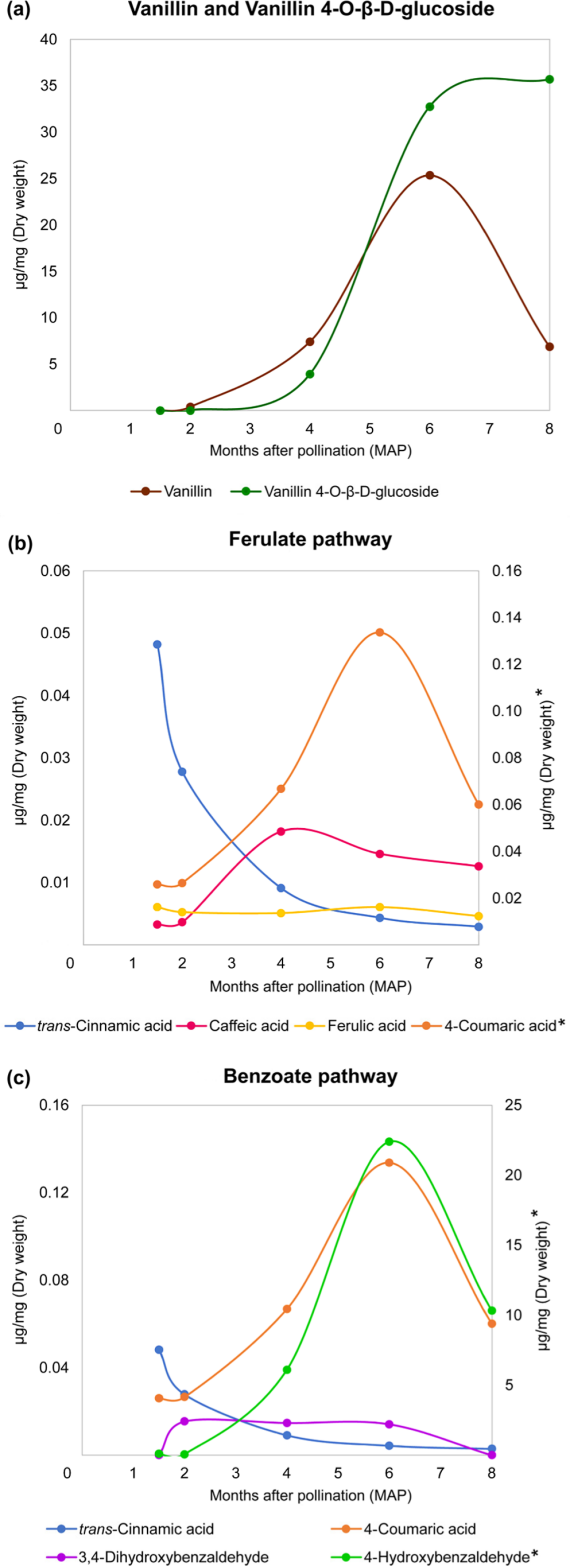
Targeted analysis of the vanillin pathways in pods at 1.5, 2, 4, 6, and 8 MAP. (a) Vanillin and vanillin 4-*O*-β-d-glucoside quantitated by HPLC-DAD. (b) Representative compounds of the ferulate pathway measured by UPLC-ESI-Q-TOF-MRM. (c) Determination of compounds in the benzoate pathway by UPLC-ESI-Q-TOF-MRM. The concentration of compounds (μg mg^–1^ DW) marked with (*) are represented on the right secondary axis of each graph.

Considering the ferulate pathway (Figure 5b), there was a notable increase in caffeic acid at four MAP, followed by a gradual decrease over successive months. Conversely, the concentration of ferulic acid remained constant throughout pod development. On the other hand, 4-hydroxybenzaldehyde, the initial precursor of the benzoate pathway, reached its peak concentration at six MAP, demonstrating a significant decrease toward the harvest period. Between the second and sixth months, the concentration of 3,4-dihydroxybenzaldehyde stayed stable. However, it was undetectable at eight MAP (Figure 5c). We also prepared analytical standard solutions of isoferulic acid, 3,4-dimethoxycinnamic acid, and 2,4-dimethoxybenzoic acid; however, none were detected in the samples.

### Comparative Metabolomic and Transcriptomic of the Vanillin Biosynthesis Pathway

To provide an overview of the vanillin biosynthesis pathway, we compared the results from both targeted and nontargeted metabolomic analyses with the expression levels of the transcripts described in each of the proposed pathways (Figure 6). The patterns of metabolite abundances were similar in both approaches. We could also annotate other compounds that are part of the pathway, which could not be quantitated due to the absence of commercial analytical standards.

**Figure 6:**
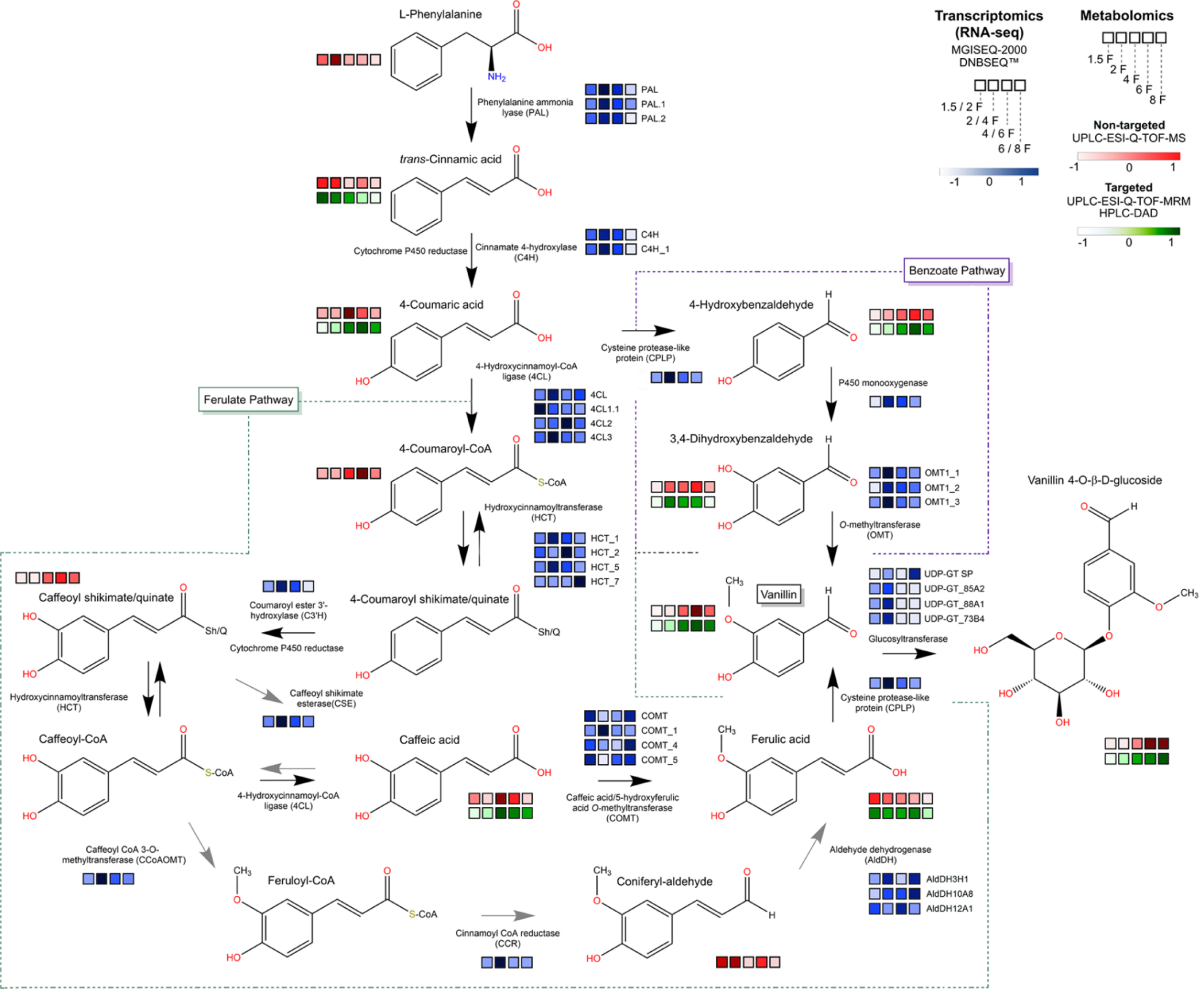
Metabolite levels and transcripts coding for enzymes putatively involved in the two proposed vanillin biosynthesis pathways in pods at 1.5, 2, 4, 6, and 8 MAP. The ferulate pathway is depicted on the left, and the benzoate pathway is shown on the right. The transcriptome heatmap scale represents transcript abundance between pairs of stages, with a white to blue color gradient indicating low and high expression levels, respectively. For the metabolomic data, the upper bar corresponds to the nontargeted method, illustrating metabolite relative abundance, where a color scale ranging from white to red indicates metabolite levels. The lower bar represents compound quantitation, displaying their concentration at each developmental stage, with a gradient color scheme from white to green. All data were based on five biological replicates.

We found a strong correspondence between the transcripts encoding a specific enzyme and the metabolic product of that enzyme (Figure 6). For instance, the initial reactions that lead to the synthesis of the precursors for either the ferulate or benzoate pathways commence with the action of phenylalanine ammonia-lyase. Our results indicate that the transcripts of genes encoding *VpPAL*, *VpPAL1*, and *VpPAL2* were up-regulated at stages 2/4F. These enzymes convert l-phenylalanine into *trans*-cinnamic acid, and the abundances of both metabolites were higher in immature pods. In the next metabolic step, *trans*-cinnamic acid is the substrate for cinnamate 4-hydrolases (*VpC4H* and *VpC4H.1*), which produce 4-coumaric acid. The transcripts encoding C4H enzymes were more abundant at stage 2/4F, while their product was more prevalent in the 4F stage.

4-Coumaric acid is a precursor for both the ferulate and benzoate pathways. In our metabolomic analyses, we found that several intermediaries of both pathways accumulate during fruit developmental stages characterized by an increase in the levels of transcripts encoding the enzymes that utilize these intermediaries as substrates (Figure 6). An example of such relationships in the benzoate pathway can be seen in the increase at stage 4 of the transcripts encoding cysteine protease-like protein (CPLP), which has 4-coumaric acid as its substrate for the synthesis of 4-hydroxybenzaldehyde. Indeed, our metabolic analyses revealed that 4-hydroxybenzaldehyde levels increase in stages 4F and 6F (Figure 6). Downstream in the benzoate pathway, a strong correspondence was observed for the synthesis of vanillin, as the higher abundance of this compound was noted at stage 6F. At the same time, the transcripts of the genes that are thought to encode the enzymes responsible for its production (*VpOMT_1*, *VpOMT_2*, and *VpOMT_3*) are up-regulated at stages 2/4F (Figure 6). However, our data suggest that ferulic acid abundance remains consistent throughout pod development, potentially indicating either rapid consumption or involvement in several metabolic pathways. Overall, the transcriptional and metabolic profiles and the quantitation obtained in this study suggest that the ferulate and the benzoate pathways are active during specific stages of fruit development.

## Discussion

Spice plant quality and economic significance are determined by their secondary metabolite composition, which is influenced by geographical factors, genotype, nutrition, and crop management. Future strategies to improve quality and yield while promoting the conservation of genetic resources depend on exploring the metabolic pathways and regulatory mechanisms that underlie the synthesis and accumulation patterns of important flavor compounds. For , some metabolomic studies have examined various plant organs. However, most of the studies have focused on a limited range of metabolites. For instance, in leaves, the temporal variation of compounds such as glucose, sucrose, acetic acid, homocitric acid, malic acid, and glucosides A and B has been studied using nuclear magnetic resonance,^50^ with some of these compounds also identified as markers for species differentiation.^51^ Furthermore, by combining LC–MS and GC–MS technologies, 127 metabolites from leaves, internodal, and aerial roots were recently identified.^52^ In this study, we annotated 778 metabolites, facilitating a distinct temporal differentiation of the pods. Our findings indicate a progressive accumulation of phenolic compounds, including vanillin and its glucoside, consistent with previous reports during the later stages of development. In contrast, young fruits exhibited high levels of glucosides, lipids, and organic acids.^35^ However, no clear temporal variation patterns were discerned for the leaf samples.

The expanded metabolite profile obtained in this study offers valuable insights into the biochemical dynamics, particularly regarding vanillin biosynthesis in during various stages of fruit development. However, elucidating the regulatory mechanisms that control the synthesis and accumulation of key compounds, especially vanillin, requires an integrative approach that combines metabolic and transcriptional data. To address this, we employed a multiomics strategy to provide a comprehensive overview of the vanillin biosynthesis pathway. This involved comparing the expression levels of transcripts coding for specific enzymes with the relative abundance of metabolites annotated through nontargeted metabolomics. Additionally, we quantitated the concentrations of metabolites directly related to vanillin using a quantitative metabolomics approach. Figure 6 illustrates the strong correspondence between three enzyme transcript sequences and their metabolic products. This corresponds to the initial reaction that leads to the synthesis of the precursors for either the ferulate or the benzoate pathways, both of which commence with the action of phenylalanine ammonia-lyase. Our results indicate that the transcripts of the genes encoding *VpPAL*, *VpPAL1*, and *VpPAL2* are up-regulated during stage 2/4F of pod development. These enzymes convert phenylalanine, which is more abundant at 2 MAP, into *trans*-cinnamic acid. The concentration of *trans*-cinnamic acid reached approximately 48 μg/g DW at 1.5 and 28 μg/g DW at 2 MAP. Its presence was only detected until 4 MAP during pod development. Interestingly, the expression level of PAL has been reported to increase throughout pod development, reaching its maximum level of around 7 MAP.^53^ Although the concentration of l-phenylalanine was not measured, PAL expression levels continuously correlated with the increasing concentration of vanillin.^32,53^ In the subsequent step of the pathway, *trans*-cinnamic acid acts as the substrate for cinnamate-4-hydroxylases (C4H) to produce 4-coumaric acid (*p*-coumaric acid). Our study identified two transcripts that putatively encode C4H enzymes, which were more abundant during stages 2 and 4 of pod development. Our targeted metabolomic analyses revealed that the concentration of 4-coumaric acid increases from 27 μg/g of DW to 134 μg/g of DW from 1.5 to 6 MAP before decreasing to 60 μg/g of DW at harvest time. This compound presumably serves as a precursor for both the ferulate and benzoate pathways. We annotated several intermediates in these pathways that accumulate during pod developmental stages. During these stages, there is a consistent increase in the levels of transcripts that encode the enzymes using these intermediates as substrates (Figure 6). In the ferulate pathway, we identified four transcript sequences that code for the enzyme 4-hydroxycinnamoyl-CoA ligase (4CL), two of which are up-regulated at early developmental stages of pods, specifically between 1.5 and 2 MAP. Additionally, one of the transcripts exhibits up-regulation during a later stage at 4/6F MAP. Rao et al.^30^ reported similar results for 4CL expression levels in specific pod tissues that are not in contact with seeds and observed higher expression levels in seeds at 2 and 2.5 MAP. Our study could not quantitate the reaction products, such as 4-coumaroyl-CoA. However, this compound was detected in our nontargeted approach and appears more abundant at 6 MAP.

Downstream in the ferulate pathway, we observed an increase in the levels of four different transcripts that putatively encode hydroxycinnamoyl transferase from stage 2 to 8 MAP. However, we were unable to detect the reaction product, 4-coumaroyl shikimate, using any of the metabolomic approaches. Moreover, we detected a transcript that putatively coded for coumaroyl ester 3′ hydroxylase (C3′ H), caffeoyl shikimate esterase, and caffeoyl CoA 3-*O*-methyl transferase (CCoAOMT) enzymes. Nevertheless, we annotated only caffeoyl shikimate/quinate, using the nontargeted approach, which was more abundant in the later stages. At the same time, the other reaction products, such as caffeoyl-CoA and feruloyl-CoA, were not detected. Nonetheless, a transcript in which the open reading frame (ORF) putatively coded for cinnamoyl CoA reductase (CCR) was detected, and its corresponding reaction product, coniferyl-aldehyde, appears to be more abundant between 1.5 and 2 MAP. *VpC3′H*, *VpCSE*, and *VpCCoAOMT* transcripts are up-regulated between 2 and 4 MAP. Furthermore, caffeic acid was highly abundant at 4 MAP. It was present throughout pod development, with a concentration ranging from 3 to 18 μg/g DW (Figure 5). We also identified four transcript sequences encoding caffeic acid/5-hydroxy ferulic acid *O*-methyltransferase (COMT). The reaction product was identified as ferulic acid, abundant at 1.5 and 6 MAP. The concentration of ferulic acid remains constant throughout pod development, averaging between 5 and 6 μg/g DW.

We also identified three transcripts in which ORFs coded for aldehyde dehydrogenases, which convert coniferyl aldehyde into ferulic acid. Compared with other compounds in the ferulate pathway, such as cinnamic acid, caffeic acid, and 4-coumaric acid, ferulic acid consistently exhibits the lowest concentrations during pod development, maintaining stable abundance levels. We identified a transcript sequence that encodes a CPLP, which has recently been reported to convert ferulic acid directly into vanillin.^14^ However, the activity of this enzyme resulted in controversy.^18^ We found that ferulic acid concentrations were inconsistent with those of vanillin during pod development. This suggests that ferulic acid may be rapidly converted into vanillin or other metabolites and utilized in various metabolic pathways. Furthermore, caffeic acid plays a role in producing C-lignin, a polymer that accumulates in the seed coat during the early stages of pod development, specifically 3 months before the emergence of vanillin.^54^ However, we observed that vanillin does not solely develop three MAP, as we measured vanillin as early as 2 MAP. This finding corresponds with the expression levels of genes involved in C-lignin biosynthesis, as reported by Chen et al.^54^ and Rao et al.^30^ Caffeic acid may be converted to caffeoyl-CoA, which is subsequently transformed to caffeoyl aldehyde through the action of CCR. Thereafter, cinnamoyl alcohol dehydrogenase converts caffeoyl aldehyde to caffeoyl alcohol. This process aids in synthesizing C-lignin polymers in the vanilla seed coat. The incorporation of caffeic alcohol into C-lignin polymers has been reported to occur exclusively in the seed coat during early pod development.^54^ Our transcriptomic analysis, in contrast to that of Rao et al.,^30^ was performed on whole pod samples rather than tissue-specific samples. This difference suggests that we may have detected remnant enzyme activity in our pooled pod samples. We annotated coniferyl aldehyde and sinapyl aldehyde in our nontargeted metabolomics analysis (see Table S5). Both compounds are involved in the synthesis of G-lignin and S-lignin, respectively. Since these types of lignin are not part of the seed coat composition,^54^ our findings indicate that these lignin types may be synthesized in the vanilla pod mesocarp. However, the mechanism by which these lignins are incorporated into the vanilla pod mesocarp remains unclear.

The benzoate pathway is a less-understood aspect of vanillin biosynthesis in vanilla pods. Our transcriptomic data regarding this pathway reveals a transcript where the ORF putatively codes for a CPLP. This protein has been previously purified and has demonstrated the ability to convert 4-coumaric acid into 4-hydroxybenzaldehyde in vitro.^16,17^ We found that *VpCPLP* or *Vp4HBS* transcript levels increase between 2 and 4 MAP. In contrast, the concentration of the reaction product, 4-hydroxybenzaldehyde (Figure 5), increases drastically, reaching concentrations 36 to 170 times higher than 4-coumaric acid throughout pod development, specifically from 2 to 8 MAP, exhibiting a pattern similar to that observed for 4-coumaric acid. Following the benzoate pathway, we identified a transcript that codes for a P450 monooxygenase, which was up-regulated during the 2/4F stage and remained active until the 4/6F stage. Its reaction product, 3,4-dihydroxybenzaldehyde, maintained a steady concentration of approximately 15 μg/g DW, similar to the corresponding transcript, throughout the intermediate stages of pod development (2–6 MAP). For the final step of the benzoate route, we measured three transcript sequences that code for *O*-methyltransferase enzymes. All these sequences showed up-regulation between 2 and 4 MAP.

In pods, the concentration of vanillin gradually increased throughout the fruit development, reaching 25 μg/mg DW at 6 MAP before drastically decreasing to around 8 μg/mg at 8 MAP. In contrast, its glucoside continued accumulating, peaking at 36 μg/mg DW during harvest (8 MAP), a trend consistent with previously reported patterns of progressive vanillin glucoside accumulation.^35,55^ Although earlier studies consistently report that vanillin is predominantly stored in a conjugated form, our findings at four MAP revealed a predominance of the free form over its glucosylated derivative. This discrepancy is normal, as our data revealed the continuous accumulation of glucovanillin toward the end of the pod maturation. The up-regulation of transcripts encoding glucosyltransferases in stages 2/4F suggests that free vanillin is rapidly glucosylated; indeed, four transcripts coding for glycosyltransferase activities were detected, one of which is a putative UDP-glycosyltransferase family 88A1 that has been previously reported to use vanillin as a preferred substrate.^56^ Contrary to the UDP-glycosyltransferase 72 family (UGT72U1) reported by Gallage et al.,^14^ evidence for a responsible gene and its accession number has not been published.

In our nontargeted metabolomics analysis, it is notable that we detected a wide variety of metabolites, even though these are putative metabolite annotations. This diversity makes elucidating the vanillin pathway a challenging task. For example, we detected benzenoid-related metabolites, including benzyl sulfate, salirepin, several BA derivatives, and benzaldehyde. These compounds have been identified in a peroxisomal β-oxidative pathway that contributes to the formation of C6–C1 aromatic volatiles from cinnamic acid in poplar following herbivory.^57,58^ Similarly, downregulation of a peroxisomal thioesterase (PhTE1) in petunia flowers^22^ suggested that flux through the phenylpropanoid pathway was increased, due to accumulation of cytosolic cinnamoyl-CoA that supports the elevated levels of phenylpropanoid products. We also identified a multitude of glucosylated compounds closely linked to the vanillin pathway, beginning with vanillic acid, which has previously been proposed as a hypothetical biosynthetic origin of *p*-hydroxybenzaldehyde from *trans*-cinnamic acid, as described by Rasmussen and Dixon^59^ and Fock-Bastide et al.^53^ Additionally, we detected compounds associated with the BA pathway, such as 4-hydroxybenzoic acid, 2,3-dihydroxybenzoic acid, and 3,4-dimethoxybenzoic acid. Other glucosylated compounds identified included *trans*-cinnamic acid-β-d-glucoside, coniferaldehyde-glucoside, 1-sinapoyl-d-glucoside, ferulic acid-4-glucoside, caffeic acid 3-glucoside, and benzoyl-d-glucoside, among others. Interestingly, UDP-glucose was present as a highly abundant metabolite throughout pod development.

The study of vanillin biosynthesis faces several challenges, including the extended time frame required for compound accumulation and the enzymatic promiscuity inherent to plants’ phenylpropanoid metabolism. Our data provide coherent profiling between transcriptomic and metabolomic data during pod developmental stages, suggesting that hydroxybenzaldehyde plays a crucial role in vanillin biosynthesis. However, despite its predominance in our sampling and experimental conditions, the benzoate pathway may not be the only one active, considering the adaptability of to the diverse environmental conditions found in the diverse habitats where it thrives.

## Supplementary Material





## Data Availability

The RNA-sequencing data were submitted to the Sequence Archive Repository of the NCBI (https://www.ncbi.nlm.nih.gov/) under BioProject no. PRJNA1240005.

## Notes

Transcriptomic profiling of *V. planifolia* leaves development (PDF)
Pathway analysis of pre-annotated compounds in *V. planifolia* pods using UPLC-ESI-QTOF-MS (XLSX)

## Abbreviations

BP: biological process
CC: cellular component
DEGs: differentially expressed genes
EDC: enhanced duty cycle
ESI: electrospray ionization
GO: gene ontology
HPLC: high-performance liquid chromatography
LC–MS: liquid chromatography–mass spectrometry
LOD: limit of detection
LOQ: limit of quantitation
MAP: months after pollination
MF: molecular function
MRM: multiple reaction monitoring
PCA: principal component analysis
QCs: quality control samples
Q-TOF: quadrupole time-of-flight
UPLC: ultraperformance liquid chromatography
*m*/*z*: mass-to-charge ratio
