# Supplementary material for: Unravelling Vanillin Biosynthesis: Integrative Transcriptomic and Metabolomic Insights into Pod Development
Source: J Agric Food Chem. 2025 Jul 17;73(30):19094–106. doi: 10.1021/acs.jafc.5c05293 (PMC12314903; doi:10.1021/acs.jafc.5c05293)
Supplement: Supplementary file 1 [file jf5c05293_si_001.pdf]

## SUPPORTING INFORMATION

# Unravelling vanillin biosynthesis: integrative transcriptomic and metabolomic insights into *Vanilla planifolia* pod development

Rebeca Hernández-Peña[1], José Luis Lorenzo-Manzanarez[2], Luis Alfredo Cruz-Ramírez[2], Delfino Reyes-López[3], Carmela Hernández-Domínguez[3], Fermín Pascual-Ramírez[4], José J. Ordaz-Ortiz[1]*

[1]Metabolomics and Mass Spectrometry Group, Unidad de Genómica Avanzada, Cinvestav, Carretera Irapuato-Léon, Km. 9.6, Libramiento Norte, C.P. 36824, Irapuato, Gto. Mexico.
[2]Molecular and Developmental Complexity Group, Unidad de Genómica Avanzada, Cinvestav, Carretera Irapuato-Léon, Km. 9.6, Libramiento Norte, C.P. 36824, Irapuato, Gto. Mexico.
[3]Facultad en Ciencias Agrícolas y Pecuarias, Benemérita Universidad Autónoma de Puebla, San Juan Acateno, Teziutlán, Puebla, C.P. 73965, Mexico.
[4]Instituto de Investigaciones en Ecosistemas y Sustentabilidad, UNAM, Campus Morelia, Morelia, Michoacán, C.P. 58190, Mexico.
(*Email: jose.ordaz.ortiz@cinvestav.mx)

**Figure S1.** Transcriptomic profiling of *V. planifolia* leaves development P. 2

**Figure S2.** Pathway analysis of pre-annotated compounds in *V. planifolia* pods using UPLC-ESI-Q-TOF-MS P. 3

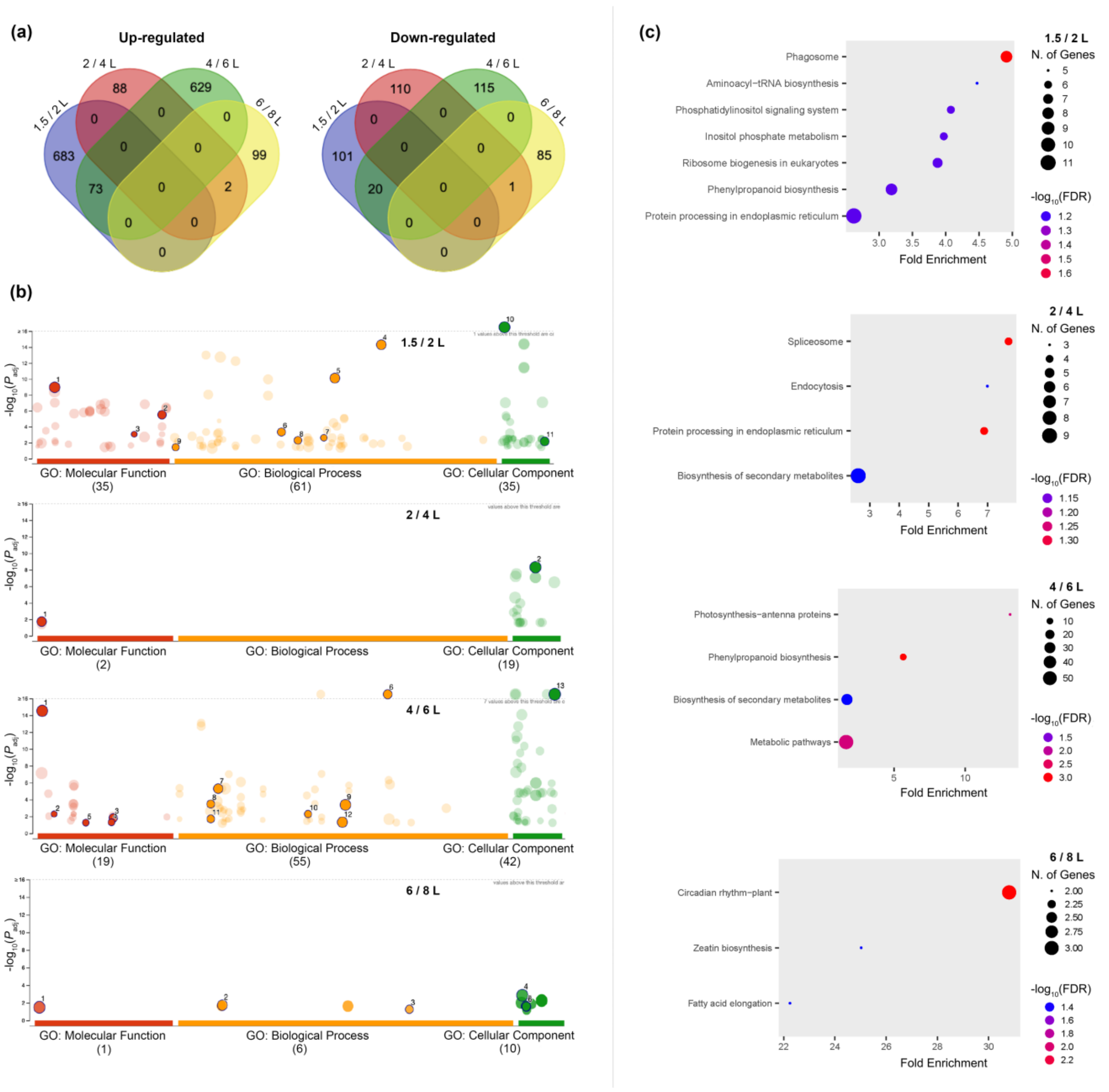


**Fig S1.** Transcriptomic profiling of *V. planifolia* leaves development a) Venn diagram illustrating the overlap of differentially expressed genes (DEGs) in *V. planifolia* leaves, including up-regulated and down-regulated genes across 1.5 / 2F, 2 / 4F, 4 / 6F, and 6 / 8F MAP stages. The numbers and circles depict the unique or overlapping DEGs that shared regulatory patterns among various stages. b) Gene ontology (GO) enrichment analysis of differentially expressed genes (DEGs) based on transcriptomic data from leaves at 1.5 / 2F, 2 / 4F, 4 / 6F, and 6 / 8F MAP. The size of the circles indicates the number of genes overrepresented in specific categories of Molecular Function (MF), Biological Process (BP), and Cellular Component (CC) in relation to Gene Ontology (GO). The numbers correspond to the IDs of the biological functions, which are detailed in Table S4. The Benjamini-Hochberg method was employed to adjust the P value (Padj) for multiple testing. c) KEGG pathway analysis of enrichment for DEGs up-regulated in leaves at 1.5 / 2F, 2 / 4F, 4 / 6F, and 6 / 8F MAP. Fold enrichment indicates the percentage of genes associated with a specific pathway. The False Dicovery Rate (FDR) illustrates the likelihood that the enrichment is due to chance.

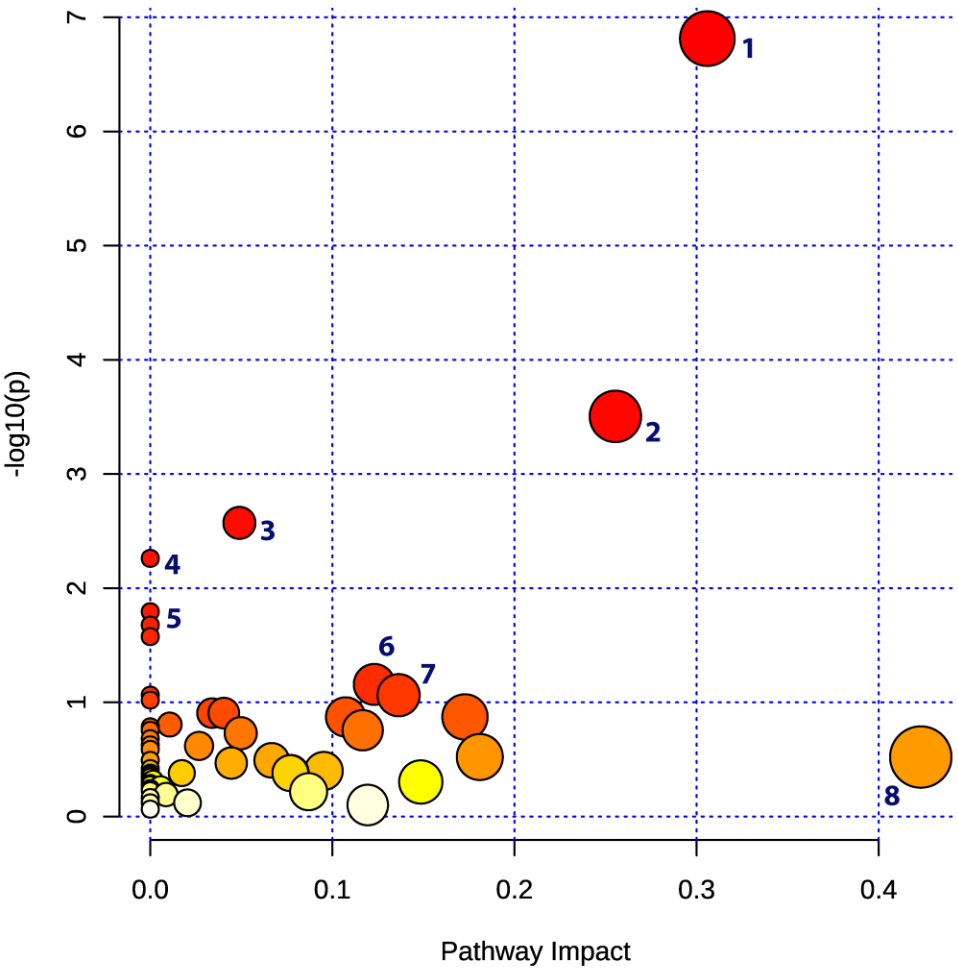


**Fig S2.** Pathway analysis of pre-annotated compounds in *V. planifolia* pods using UPLC-ESI-Q-TOF-MS. 1. Phenylpropanoid biosynthesis; 2. Alanine, aspartate, and glutamate metabolism; 3. Carbon fixation in photosynthetic organisms; 4. Nicotinate and nicotinamide metabolism; 5. Glycine, serine, and threonine metabolism; 6. Tyrosine metabolism; 7. Starch and sucrose metabolism; 8. Phenylalanine metabolism. Colour intensity represents -log10(p) and circle size correlates with pathway impact value.
